# Diabetes websites lack information on dietary causes, risk factors, and preventions for type 2 diabetes

**DOI:** 10.3389/fpubh.2023.1159024

**Published:** 2023-07-13

**Authors:** Lisa T. Crummett, Muhammad H. Aslam

**Affiliations:** Life Sciences, Soka University of America, Aliso Viejo, CA, United States

**Keywords:** diabetes, risk factors, causes, prevention, websites, diet, nutrition

## Abstract

**Introduction:**

Type 2 diabetes (T2D) is a growing public health burden throughout the world. Many people looking for information on how to prevent T2D will search on diabetes websites. Multiple dietary factors have a significant association with T2D risk, such as high intake of added sugars, refined carbohydrates, saturated fat, and red meat or processed meat; and decreased intake of dietary fiber, and fruits/vegetables. Despite this dietary information being available in the scientific literature, it is unclear whether this information is available in gray literature (websites).

**Objective:**

In this study, we evaluate the use of specific terms from diabetes websites that are significantly associated with causes/risk factors and preventions for T2D from three term categories: (A) dietary factors, (B) nondietary nongenetic (lifestyle-associated) factors, and (C) genetic (non-modifiable) factors. We also evaluate the effect of website type (business, government, nonprofit) on term usage among websites.

**Methods:**

We used web scraping and coding tools to quantify the use of specific terms from 73 diabetes websites. To determine the effect of term category and website type on the usage of specific terms among 73 websites, a repeated measures general linear model was performed.

**Results:**

We found that dietary risk factors that are significantly associated with T2D (e.g., sugar, processed carbohydrates, dietary fat, fruits/vegetables, fiber, processed meat/red meat) were mentioned in significantly fewer websites than either nondietary nongenetic factors (e.g., obesity, physical activity, dyslipidemia, blood pressure) or genetic factors (age, family history, ethnicity). Among websites that provided “eat healthy” guidance, one third provided zero dietary factors associated with type 2 diabetes, and only 30% provided more than two specific dietary factors associates with type 2 diabetes. We also observed that mean percent usage of all terms associated with T2D causes/risk factors and preventions was significantly lower among government websites compared to business websites and nonprofit websites.

**Conclusion:**

Diabetes websites need to increase their usage of dietary factors when discussing causes/risk factors and preventions for T2D; as dietary factors are modifiable and strongly associated with all nondietary nongenetic risk factors, in addition to T2D risk.

## Introduction

Type 2 diabetes (T2D) is a chronic disease characterized by excessive levels of glucose in the blood resulting from the cells’ inability to respond to insulin, termed insulin resistance, and an inability of pancreatic beta cells to produce adequate levels of insulin. The International Diabetes Federation (IDF) has stated that the increasing global prevalence of T2D presents a large social, financial, and health system burden across the world (1). The global prevalence of T2D in 20–79 year old adults was estimated at 10.5% in 2022 and it is projected to increase to 12.2% by 2045 (2). A staggering 38% of the adult population in the United States (US) has prediabetes (insulin resistance) and 11.3% have T2D (3). “Adult-onset diabetes” was a commonly used term for T2D because it only affected adults; but that is no longer true. In 2021, there were approximately 41,600 new cases of T2D diagnosed in children, worldwide (4). Considering that only the proximate cause of death appears on death certificates, diabetes-associated deaths may be significantly underestimated (5).

Understanding what factors promote the development of insulin resistance and T2D is imperative in curbing the soaring T2D pandemic. Genetic risk factors for T2D include family history (6), advanced age (2), and non-white ethnicity (7). However, ethnic groups that show a relatively high prevalence of T2D in the US, show a much lower T2D prevalence in their country of origin (7); showing that environmental factors play a critical role in T2D risk. Further, in order to explain the rapid increase in T2D prevalence over the last three decades (8, 9), and reduce the predicted growth of T2D prevalence in the future (2, 8), we need to look beyond genetic (non-modifiable) risk factors for T2D, and focus on dietary and lifestyle-associated risk factors that are modifiable.

A review of 86 meta-analyzes that analyzed risk factors for T2D reported “convincing evidence” for the association between T2D risk and the following modifiable risk factors: low whole grain consumption, metabolically healthy obesity, increased sedentary time, low adherence to a healthy dietary pattern, high level of serum uric acid, which has been associated with high fructose/sugar intake (10–12), low level of serum vitamin D, and decreased conscientiousness (13). A meta-analysis of cohort studies that specifically examined lifestyle-related risk factors for T2D reported the following high-risk factors: obesity, (especially central obesity), metabolic syndrome components (hypertension, dyslipidemia), lack of physical activity, high consumption of sugar-sweetened beverages, processed red meat, refined grains, and alcohol; and low consumption of fruits, vegetables, fiber, and whole grains (14). It is important to note that these dietary risk factors were still significantly associated with T2D after controlling for body mass index (BMI) (14).

Given that dietary factors may be the most amenable to modification to prevent the development of T2D, some recent meta-analyzes of T2D risk factors have focused solely on dietary factors. One study reported significant associations with T2D risk and higher intake of cereal fiber, unsaturated fatty acids, magnesium, and polyphenols; and reduced glycemic load, intake of added sugars, and intake of high-sugar beverages (15). Another study reported a significant negative association with T2D risk and intake of foods associated with a Mediterranean diet, including: whole grains, low-fat dairy products, yogurt, olive oil, chocolate, fiber, magnesium, and flavonoids (16). The same study reported a significant positive association with T2D risk and high glycemic index/load diets, high consumption of red and processed meat, sugar, and artificial sugar-sweetened beverages (16).

To understand how to prevent the development of T2D, many people are likely to search diabetes websites, *via* a Google search, rather than searching scientific journal articles. Based on searching a limited number of diabetes websites, we found limited information on nongenetic (lifestyle-associated) causes/risk factors or preventions for T2D, aside from obesity and lack of physical activity. The observed lack of information on diabetes websites, regarding nongenetic T2D causes/risk factors and preventions, may be anecdotal, or it may point to a real lack of information in the gray literature. To answer this important public health question, we used web scraping and computer code to extract and quantify terms associated with T2D causes/risk factors and preventions from 73 diabetes websites. We quantified the use of terms for T2D causes/risk factors and preventions that were categorized as (A) dietary factors (B) nondietary nongenetic (lifestyle-associated) factors, and (C) genetic (non-modifiable) factors. We determined if term category (dietary, nondietary nongenetic, genetic) or website type (business, government, nonprofit) had a significant effect on the percentage of sites providing specific terms.

## Materials and methods

### Website selection

**Figure 1:**
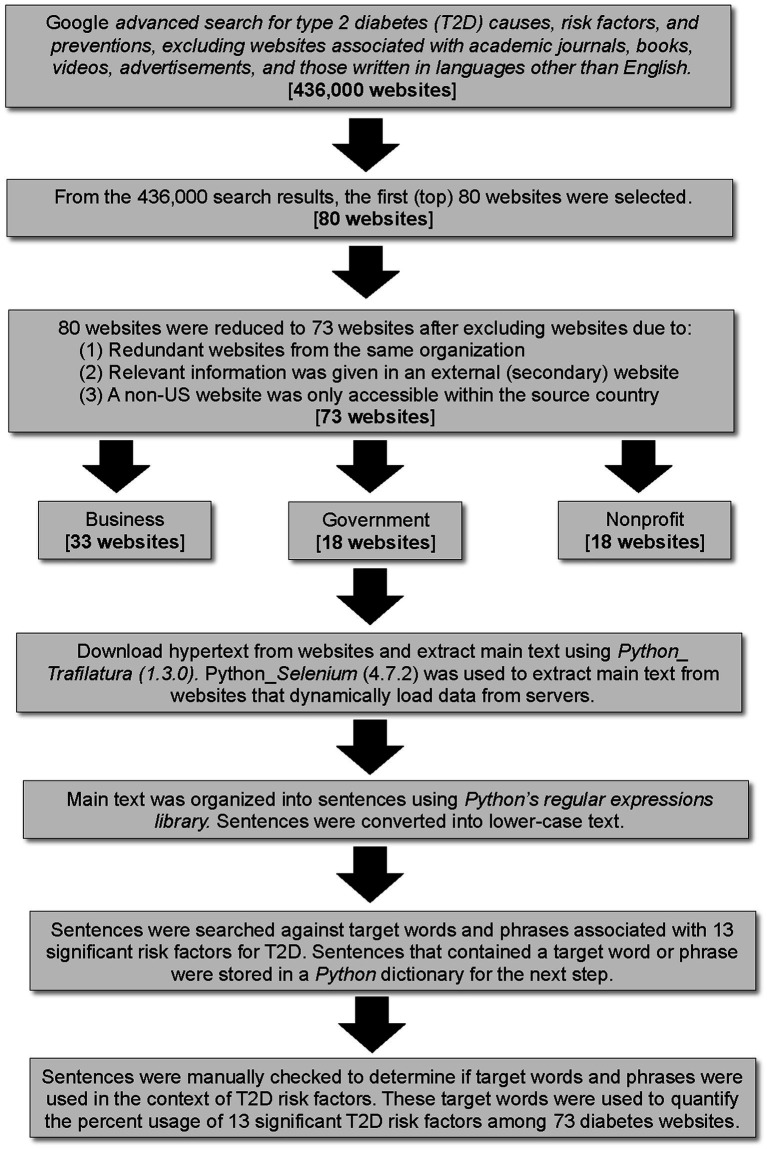
Flow diagram illustrating methodology for website selection, categorization, web scraping, sentence filtering, and quantification of terms. Software package versions are indicated in parentheses.

In November 2022, we performed a “Google Advanced Search” to search for diabetes websites to analyze. We used the following advanced search criteria, which resulted in 436,000 websites in the search engine results (Figure 1): *All these words: “type 2 diabetes”; this exact word or phrase: “causes” OR “risk” OR “prevention”; none of these words: “pubmed, “doi:,” “download pdf,” “youtube,” “amazon,” “advertisement”; Language: English; Region: anywhere; Last update: past year; site or domain: NA; terms appearing: In the text of the page; file type: any format; usage rights: not filtered by license.* The “none of these words” criteria effectively excluded websites associated with academic journals, books, videos, and advertisements. The first 80 websites in the search engine results were selected for analysis (Figure 1). Other studies in which website data from a keyword search were analyzed, selected the first 50–60 websites that appeared in the search engine results (17–20). Eighty websites were reduced to 73 websites after excluding some sites due to one of the following reasons: (1) website was funded by the same organization/company as that of another included website, or (2) one had to click on several links to access relevant information (the information was external to the website), or (3) the site was outside the US and was only accessible within the source country (Figure 1).

### Categorization of websites

The 73 websites that were included in the analysis were categorized as business, government, or nonprofit (Supplementary Table S1; Figure 1). Websites owned by for-profit companies were assigned to the business category (*n* = 33) and most had .com domains (Supplementary Table S1). Websites owned by county, state, or federal government were assigned to the government category (*n* = 18) and most had .gov domains (Supplementary Table S1). Websites owned by tax-exempt organizations, either nonprofit or not-for-profit (their income is not distributed to members or officers of the organization), were assigned to a single “nonprofit” category (*n* = 22) (Supplementary Table S1). All nonprofit websites had a .org domain, except for one website, which had a .edu domain (Supplementary Table S1). For each of the 73 websites, the site type, source country, and SEMrush score (measurement of a website’s popularity) were recorded (Supplementary Table S1). Sixty websites were from the US, nine were from Non-US countries, and four were international (Supplementary Table S1).

### Selection of target words and phrases

We selected target words and phrases to quantify from websites that were associated with statistically significant risk factors for T2D (6, 7, 13–16) and we categorized them into three term categories: (A) dietary factors, (B) nondietary nongenetic (lifestyle-associated) factors, and (C) genetic (non-modifiable) factors (Table 1). Dietary words and phrases were further categorized into the following six subcategories: (1) sugar, (2) refined carbohydrates, (3) fiber, (4) fruits or vegetables, (5) dietary fat, and (6) red meat or processed meat (Table 1). Nondietary nongenetic (lifestyle-associated) words and phrases were further categorized into the following four subcategories: obesity, physical activity, blood pressure, dyslipidemia (Table 1). Genetic (non-modifiable) words and phrases were further categorized into three subcategories: age, family history, and ethnicity. All target words and phrases were present in at least three of the 73 websites (Table 1).

**Table 1:** Target words and phrases that were quantified from 73 diabetes websites and their categorization for statistical analyzes.

Term Category	Term subcategory	Target words and phrases in term subcategory				
Category A: Dietary Factors	Sugar	Sugar_	Sweet_	Soda	Sucrose	
	Refined carbs	Glycemic index	Glycemic load	Processed	Refined	
	Fiber	Fiber	Whole grain /whole-grain			
	Fruit/vegetables	Fruit	Vegetables			
	Dietary fat	Saturated fat_	Unsaturated fat_	Low-fat/low fat		
	Red meat or processed meat	Red meat	Processed meat			
Category B: Nondietary Nongenetic Factors	Obesity	Obese/obesity	Weight_	Abdominal/belly fat	Body mass index/BMI	Adiposity
	Physical activity	Exercis_	Activ_	Sedentary		
	Blood pressure	Hypertension	Blood pressure			
	Dyslipidemia	Dyslipidemia	Triglyceride	Cholesterol	HDL	LDL
Category C: Genetic Factors	Age	Age_	Old_			
	Family history	History	Genetic_			
	Ethnicity	Ethnicit_				

All target words and phrases in the same row are associated with the same “term subcategory” (second column). The use of “_” after a word denotes that any word containing that root word was counted (e.g., “weight_” includes “overweight,” “weight gain,” “extra weight,” and “healthy weight”).

### Web scraping, sentence filtering, and quantification of terms

We downloaded the hypertext from each of the 73 websites and then used a Python package, Trafilatura (version 1.3.0) (21), to extract the main text of each website. We used Python’s web-automating library, Selenium (version 4.7.2) (22), to extract the complete main text from websites that dynamically load data from servers. The main text was organized into sentences using Python’s built-in regular expressions library. Sentences were converted to lower-case text before searching for target words and phrases. Each sentence was searched against target words and phrases (Table 1). Sentences containing a target word or phrase were stored in a Python dictionary. Each sentence in the Python dictionary was manually checked to determine if a target word or phrase was being used in the context of causes/risk factors or preventions for T2D. For example, the sentence “Avoid sugary drinks,” was recorded as providing a “sugar” term. However, the sentence “Patients with type 2 diabetes have high blood sugar levels,” would be deleted from the Python dictionary, because sugar was not used in a dietary context as a cause/risk factor or prevention for T2D. A flow diagram of the above methodology is provided (Figure 1).

All the sentences that passed the manual filtering step were used to quantify per-website count of target words and phrases (Table 1); and those counts were used to determine subcategory and category usage (Table 1) among 73 websites (Table 1). The computer code that we wrote for website text extraction and quantification of target words and phrases is available on GitHub: https://github.com/MHannanAslam/Quantification-of-T2DM-risk-factors-from-websites (23).

### Statistical analysis

We quantified the presence/absence (1/0) of at least one target word or phrase associated with each of the 13 subcategory terms (Table 1), per website. The presence/absence values were used to calculate the percentage of websites that used at least one target word or phrase associated with each of the 13 subcategory terms (Table 1). We took this approach because some subcategory terms, such as “sugar,” have more synonymous words and phrases associated with them than other subcategory terms, and therefore, if we had quantified the total number of target words and phrases used per subcategory term, per website, it would have yielded inflated counts for subcategory terms that have a lot of synonyms.

To determine the effect of term category (dietary, nondietary nongenetic, genetic) and website type (business, government, nonprofit) on the usage of words and phrases associated with 13 subcategory terms (Table 1), among 73 websites (Supplementary Table S1), a repeated measures general linear model was performed in SPSS Statistics (version 28.0.1.1). In our model, the 13 subcategory terms (sugar, refined carbohydrates, fiber, fruit/vegetables, dietary fat, red meat/processed meat, obesity, physical activity, blood pressure, dyslipidemia, age, family history, ethnicity) were the subjects; the percentage of websites that provided at least one word or phrase associated with a given subcategory term (Table 1) was the response variable (repeated measure); the term category (dietary, nondietary nongenetic, genetic) was the between subjects factor; and website type (business, government, nonprofit) was the within subjects variable. A Bonferroni correction was applied to multiple pairwise comparisons of website types and term category types. Statistical significance was set to *p* < 0.05.

## Results

### Term category vs. percent usage of specific terms

**Figure 2:**
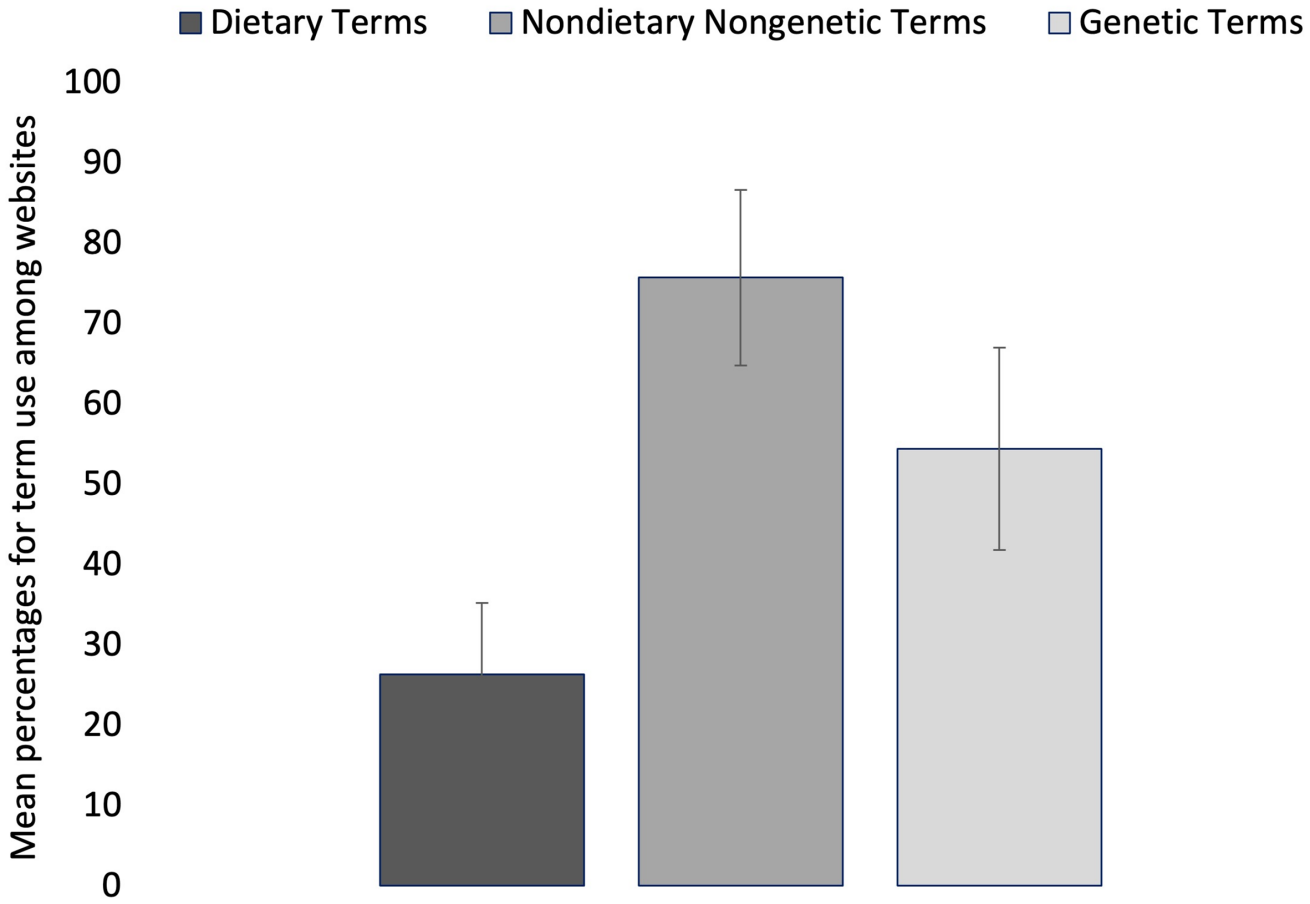
Mean percentages for term use among 73 diabetes websites. Terms are grouped into three categories: dietary, nondietary nongenetic (lifestyle-associated), and genetic (non-modifiable). Error bars represent mean standard error for the term percentages within each category.

Among the 73 websites analyzed that discussed T2D causes/risk factors and preventions, term category (dietary, nondietary nongenetic, genetic) had a significant main effect on the usage of specific terms (*p* = 0.016; Figure 2). Mean percent usage of dietary terms (26.3% ± 8.9% SE) was significantly lower (*p* = 0.016) than mean percent usage of nondietary nongenetic terms (75.7% ± 10.9% SE) and nonsignificantly lower (*p* = 0.292) than mean percent usage of genetic terms (54.4% ± 12.6% SE) (Figure 2). The mean percent usage of nondietary nongenetic terms (75.7% ± 10.9% SE) was not significantly different (*p* = 0.685) than the mean percent usage of genetic terms (54.4% ± 12.6% SE) (Figure 2).

**Figure 3:**
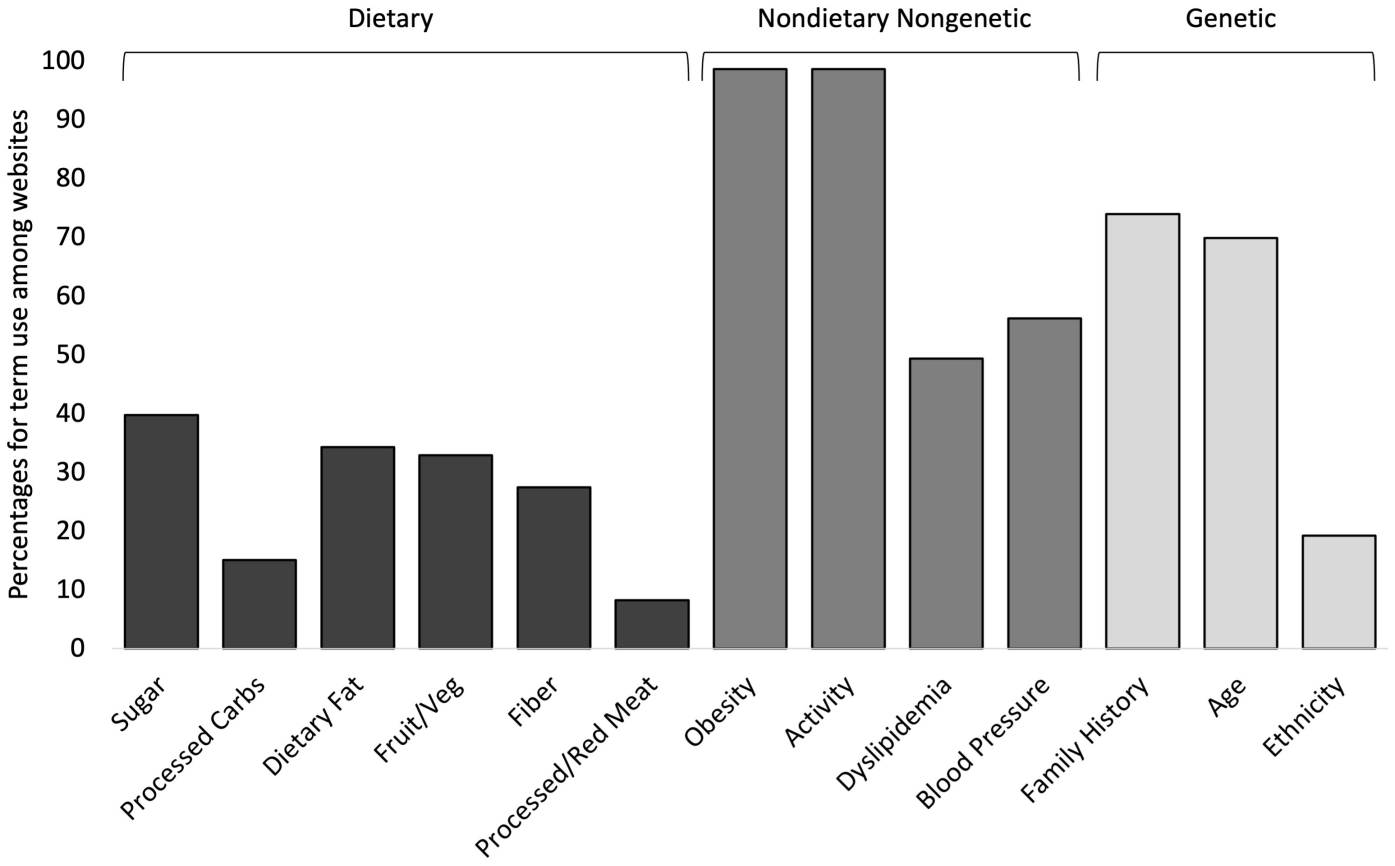
Percentages for term use among 73 diabetes websites for 13 subcategory terms. The three term categories used in statistical analyzes are shown above the brackets at the top of the figure.

When examining the use of specific terms among websites as causes/risk factors and preventions for T2D, obesity and physical activity were discussed in nearly all of the websites (98.6% for each term); age and family history were discussed in most websites (69.8 and 74.0% respectively); blood pressure and dyslipidemia were discussed in approximately half of the websites (56.2 and 49.3% respectively); and each of the six dietary terms were discussed in less than 40% of websites, with sugar being the most mentioned (39.7%) of the dietary terms and processed meat/red meat being the least mentioned (8.2%) (Figure 3).

### Website type vs. percent usage of specific terms

**Figure 4:**
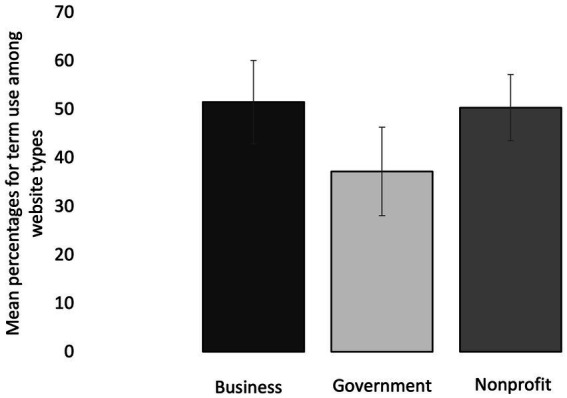
Mean percentages for term use (all term categories included) among 73 diabetes websites that are grouped into three type categories: business, government, and nonprofit. Error bars represent mean standard error for the term percentages within each website category.

**Figure 5:**
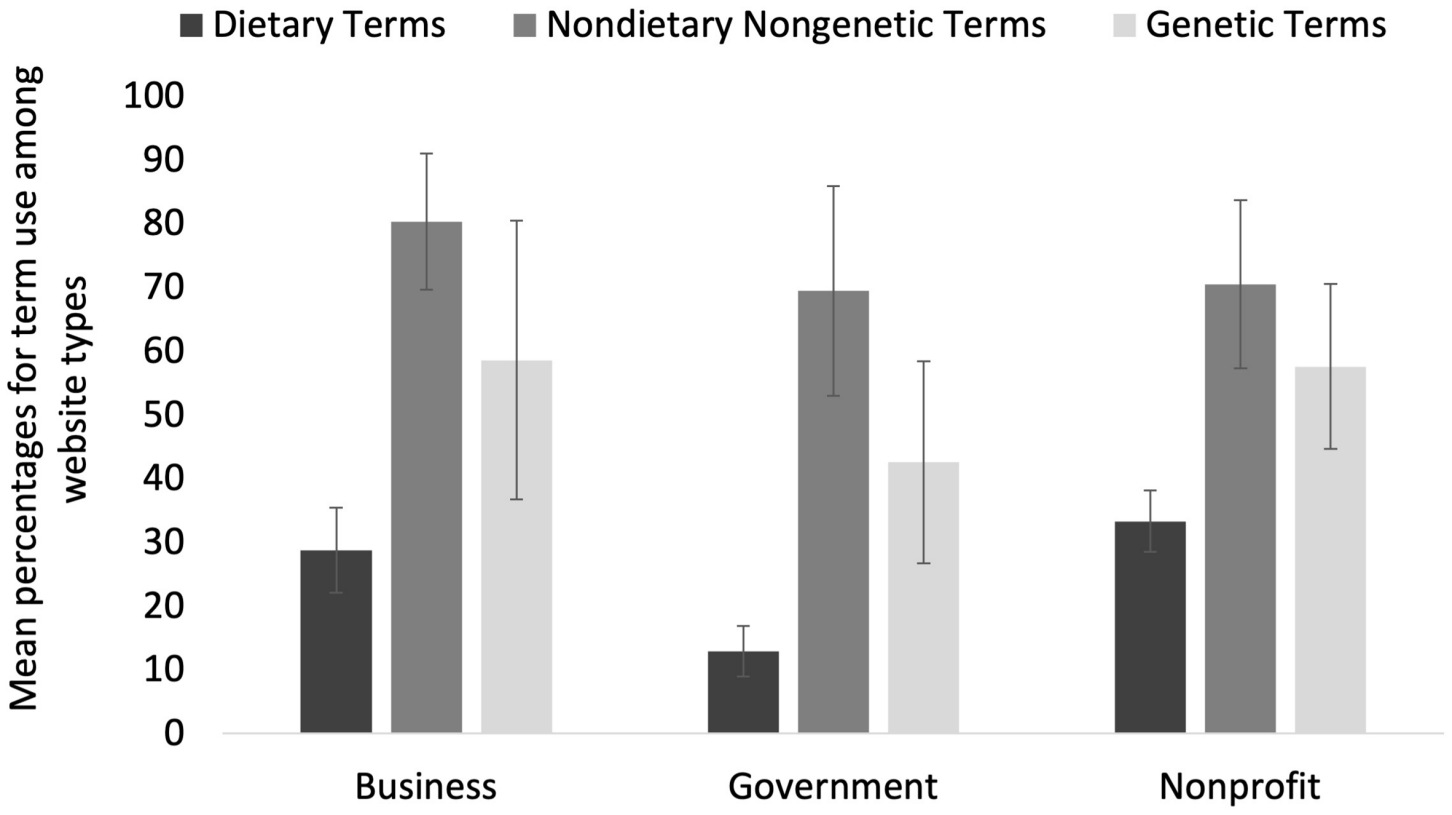
Mean percentages for term use among 73 websites, where terms are grouped into three subcategories: dietary, nondietary nongenetic (lifestyle-associated), and genetic (non-modifiable) and websites are grouped into three type categories: business, government, and nonprofit. Error bars represent mean standard error for the term percentages within each category.

Website type (business, government, nonprofit) had a significant main effect on the usage of specific terms (*p* < 0.001; Figure 4) for causes/risk factors and preventions for T2D. The mean percent usage of terms (all categories) was significantly lower among government websites (37.2% ± 9.1% SE) compared to business websites (51.5% ± 8.6% SE; *p* = 0.009) and compared to nonprofit websites (50.3% ± 6.8% SE; *p* = 0.007) (Figure 4). There was no significant difference (*p* = 1.000) in mean percent term usage between business websites (51.5% ± 8.6% SE) and nonprofit websites (50.3% ± 6.8% SE) (Figure 4). The interaction between term category x website type was not significant (*p* = 0.133) in its effect on percent term usage; meaning that the effect of term category on percent term usage was not significantly different among different website types (Figure 5).

### “Healthy diet” used without mention of dietary terms

**Figure 6:**
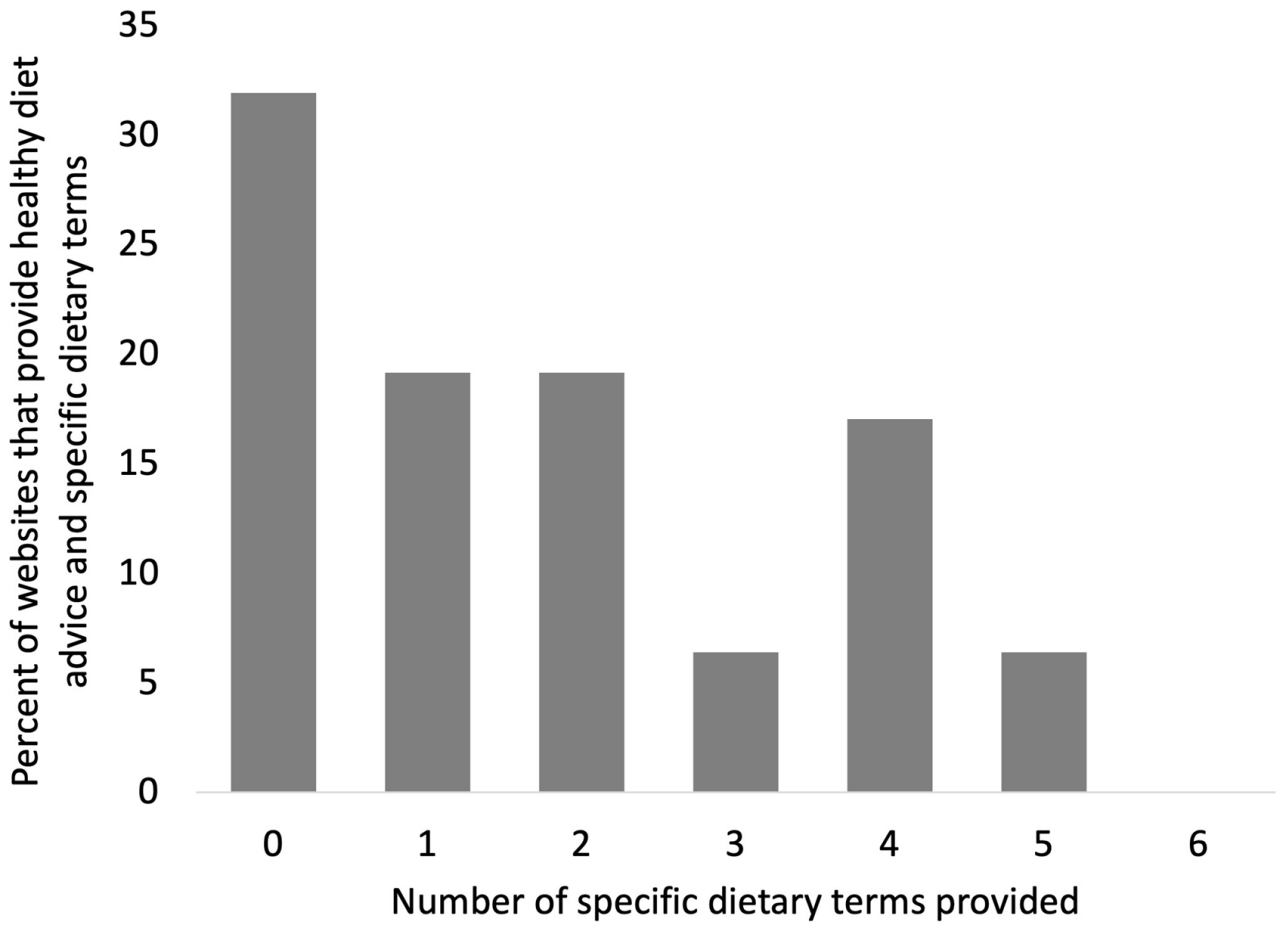
Of the 47 diabetes websites that provide “healthy diet advice,” this is the percentage of websites that provide a given number of dietary terms. The total number of dietary terms quantified from diabetes websites was six.

We observed the use of nonspecific dietary guidance in some websites, including “healthy diet” (27 websites), “healthy eating” (19 websites), “eat healthy” (12 websites), and “balanced diet” (4 websites), but in many cases, no specific dietary terms (sugar, processed carbs, fiber, etc.) that are associated with significant causes/risk factors and preventions for T2D were provided in addition to the nonspecific dietary guidance. Among the 47 websites that provided some form of nonspecific dietary guidance, 32% did not provide any dietary terms associated with significant causes/risk factors and preventions for T2D, 19% provided only one dietary term, and 19% provided only two specific dietary terms (Figure 6). Thus, only 30% of the websites providing “eat healthy” advice for causes/risk factors and preventions for T2D, also provided more than two specific dietary terms with the advice (Figure 6).

## Discussion

### Significance of findings

To our knowledge, a systematic review of causes/risk factors and preventions for T2D in the gray literature (websites) has not been performed until now. There is a great need for this type of review because of the growing prevalence of T2D around the world and the need for accurate information about T2D prevention to be accessible to everyone. An unexpected finding was that government websites, comprised of mostly state and federal government agencies (88%), provided significantly fewer causes/risk factors and preventions for T2D, from all categories, than either business websites or nonprofit websites. This suggests that government agencies need to invest more effort into ensuring that the health information provided in their diabetes websites is up-to-date, informative, and effective in disease prevention. We were also surprised to find that many of the websites that provide “eat healthy” guidance in association with causes/risk factors and preventions for T2D, provided little to no specific dietary guidance, such as: limit intake of added sugars (13–16), increase intake of polyunsaturated fats (16, 24, 25), increase intake of high-fiber foods (14–16, 26, 27). Without such dietary guidance, phrases like “eat healthy” are not useful in educating the public on how to prevent T2D. Since we only examined websites written in English, our findings cannot be extrapolated to diabetes websites written in other languages and we encourage others to conduct similar studies of diabetes websites written in non-English languages. Overall, our findings demonstrate a paucity of information on diabetes websites regarding dietary causes/risk factors, and preventions for T2D. This lack of dietary information is problematic for several reasons, which we will discuss below.

### Why exclusion of dietary risk factors and preventions for T2D is problematic

All four of the nondietary nongenetic (lifestyle-associated) risk factors for T2D (obesity, physical activity, dyslipidemia, and high blood pressure) are significantly associated with dietary factors, which may be their ultimate cause. An example of a strong association between a dietary risk factor and multiple nondietary nongenetic (lifestyle-associated) risk factors, is found with sugar. In addition to promoting insulin resistance (28–30), high sugar and/or fructose intake has been shown to promote dyslipidemia and increase fat storage (31–33), increase visceral adiposity (28, 33, 34), and cause leptin resistance (33, 35, 36), which is associated with (1) an increased drive to consume excessive amounts of energy (37) and (2) a reduction in energy expenditure (associated with lethargy) (32, 38). Further, many studies have shown that high sugar intake, particularly the fructose component of sugar, promotes hypertension (39–42), *via* stimulation of the sympathetic nervous system. This illustrates how a dietary risk factor for T2D is strongly associated with all the nondietary nongenetic risk factors: obesity, physical activity, dyslipidemia, and blood pressure; and thus, should be included.

When discussing obesity in association with T2D, it is important to point out that certain dietary factors promote an increase in different types of body fat, and not all body fat is associated with T2D. Subcutaneous fat is not a significant predictor of T2D whereas visceral fat (aka central or abdominal fat) is the strongest predictor of insulin resistance and T2D incidence (43–47). There are significant dietary associations with visceral fat. Overfeeding normal individuals with saturated fat resulted in a two-fold higher increase in visceral fat (the dangerous fat), compared to overfeeding with polyunsaturated fats (48). Studies also show that replacing saturated fat with polyunsaturated fats significantly improves insulin sensitivity (24, 25). Excess fructose intake has been shown to significantly increase visceral fat, but not subcutaneous fat, and significantly decreases insulin sensitivity (28). Alternately, excess glucose intake significantly increases subcutaneous fat, but not visceral fat, and does not affect insulin sensitivity (28). Similarly, when sugar (fructose + glucose) was replaced with starch (glucose) in children with obesity and metabolic syndrome, their glucose tolerance significantly improved in just nine days, along with their blood pressure, triglycerides, and insulin levels (30). Thus, information regarding specific dietary modifications, particularly reduced intake of added sugars and replacement of saturated fats with polyunsaturated fats, is important for reducing visceral adiposity and associated T2D risk.

Among lifestyle factors that can be modified to prevent the development of T2D, modification of dietary factors, alone, may be just as effective at reducing T2D risk as increasing physical activity, alone. A recent meta-analysis of randomized controlled trials and observational studies examining dietary factors and T2D risk, reported that dietary interventions, with or without physical activity, significantly decreased T2D risk in both high risk populations and the general population (16). People with T2D who changed their diet to a paleolithic diet over 12 weeks, showed significant improvement in glycemic control and insulin sensitivity; and the improvement was not significantly different than participants who underwent an exercise intervention + dietary intervention (49). Other intervention studies reported similar findings, in that diet + exercise intervention did not show greater improvement of glycemic control and insulin sensitivity than diet intervention alone (50, 51). For all the reasons that we have discussed, diabetes websites should make a concerted effort to include significant dietary factors when discussing T2D causes/risk factors and preventions to better educate the public on how to prevent the development of T2D.

## Data availability statement

The raw data supporting the conclusions of this article will be made available by the authors, without undue reservation.

## Author contributions

LC conceptualized the study and designed the methodology, performed data curation, statistical analysis, and interpretation of the data, and prepared the first draft. MA wrote computer code for data extraction and performed data curation. MA and LC revised subsequent drafts and read and approved the final manuscript. All authors contributed to the article and approved the submitted version.

## Conflict of interest

The authors declare that the research was conducted in the absence of any commercial or financial relationships that could be construed as a potential conflict of interest.

## Publisher’s note

All claims expressed in this article are solely those of the authors and do not necessarily represent those of their affiliated organizations, or those of the publisher, the editors and the reviewers. Any product that may be evaluated in this article, or claim that may be made by its manufacturer, is not guaranteed or endorsed by the publisher.
