# Supplementary material for: Diabetes websites lack information on dietary causes, risk factors, and preventions for type 2 diabetes
Source: Front Public Health. 2023 Jul 13;11:1159024. doi: 10.3389/fpubh.2023.1159024 (PMC10373935; doi:10.3389/fpubh.2023.1159024)
Supplement: Supplementary file 1 [file Table_1.docx]

Table S1. Website profile information from 73 websites that provide information on type 2 diabetes causes/risk factors and preventions. The “SEMr” column gives the SEMrush score for each website, which is a compound metric that represents a website’s overall quality, popularity, and search engine optimization performance; sites with lower SEMrush scores outperform sites with higher SEMrush scores. The “Site Type” columns categorizes each website as either Business, Govern (Government), or Nonprofit (includes both Nonprofit and Not-For-Profit). The “Country” column gives 3-letter country codes (AUS = Australia, CAN = Canada, DEU = Germany, GBR = United Kingdom of Great Britain and Northern Ireland, IND = India, USA = United States of America) and “Int” = International.

| **Website Url** | **SEMr** | **Site Type** | **Country** |
| --- | --- | --- | --- |
| https://www.mayoclinic.org/diseases-conditions/type-2-diabetes/symptoms-caUSAes/syc-20351193 | 20 | Nonprofit | USA |
| https://www.britannica.com/science/diabetes-mellitUSA | 21 | Business | USA |
| https://www.healthline.com/nutrition/prevent-diabetes | 27 | Business | USA |
| https://www.niddk.nih.gov/health-information/healthy-moments/episodes/reducing-your-risk-for-type-2-diabetes | 36 | Govern | USA |
| https://www.cdc.gov/diabetes/prevent-type-2/guide-prevent-type2-diabetes.html | 44 | Govern | USA |
| https://my.clevelandclinic.org/health/diseases/22206-insulin-resistance | 49 | Nonprofit | USA |
| https://medlineplUSA.gov/ency/article/001214.htm | 73 | Govern | USA |
| https://www.hopkinsmedicine.org/health/conditions-and-diseases/diabetes/type-2-diabetes | 109 | Nonprofit | USA |
| https://www.health.ny.gov/diseases/conditions/diabetes/prediabetes/ | 142 | Govern | USA |
| https://www.goodrx.com/conditions/diabetes-type-2 | 167 | Business | USA |
| https://www.hsph.harvard.edu/nutritionsource/disease-prevention/diabetes-prevention/preventing-diabetes-full-story/ | 175 | Nonprofit | USA |
| https://www.drugs.com/health-guide/type-2-diabetes-mellitUSA.html | 177 | Business | USA |
| https://www.verywellhealth.com/ask-an-expert-risk-factors-type-2-diabetes-5521615 | 191 | Business | USA |
| https://on.nyc.gov/type2diabetes | 199 | Govern | USA |
| https://kidshealth.org/en/parents/type2.html | 278 | Nonprofit | USA |
| https://www.cdhd.wa.gov/health/diabetes | 345 | Govern | USA |
| https://healthy.kaiserpermanente.org/washington/health-wellness/healtharticle.lower-your-risk-of-type-2-diabetes | 363 | Nonprofit | USA |
| https://www.who.int/news-room/fact-sheets/detail/diabetes | 373 | Nonprofit | Int |
| https://www.uptodate.com/contents/type-2-diabetes-overview-beyond-the-basics | 448 | Business | USA |
| https://health.maryland.gov/phpa/ccdpc/diabetes/pages/prevention.aspx | 667 | Govern | USA |
| https://portal.ct.gov/DPH/Health-Education-Management--Surveillance/Diabetes/General-Information-about-Diabetes | 668 | Govern | USA |
| https://www.merckmanuals.com/en-ca/home/hormonal-and-metabolic-disorders/diabetes-mellitUSA-dm-and-disorders-of-blood-sugar-metabolism/diabetes-mellitUSA-dm | 934 | Business | USA |
| https://www.self.com/story/type-2-diabetes-cAUSAses | 935 | Business | USA |
| https://www.betterhealth.vic.gov.au/health/conditionsandtreatments/diabetes-type-2#risk-factors-for-type-2-diabetes | 966 | Govern | AUS |
| https://www.cigna.com/knowledge-center/diabetes | 1089 | Business | USA |
| https://www.hoUSAtonmethodist.org/blog/articles/2022/nov/how-do-you-know-if-you-have-diabetes/ | 1404 | Nonprofit | USA |
| https://khealth.com/learn/diabetes/type-2-diabetes/ | 1516 | Business | USA |
| https://dpbh.nv.gov/Programs/Diabetes/dta/FAQs/Diabetes_-_FAQs/ | 1679 | Govern | USA |
| https://healthandwelfare.idaho.gov/health-wellness/diseases-conditions/diabetes | 2120 | Govern | USA |
| https://www.ama-assn.org/delivering-care/diabetes/what-doctors-wish-patients-knew-about-type-2-diabetes-prevention | 2220 | Nonprofit | USA |
| https://www.montgomerycountymd.gov/healthymontgomery/programs/type-2-diabetes/index.html | 2900 | Govern | USA |
| https://www.optum.com/health-articles/article/health-conditions/what-do-i-need-know-about-type-2-diabetes/ | 2940 | Business | USA |
| https://publichealth.sccgov.org/disease-information/diabetes/tips-prevent-and-manage-type-2-diabetes/know-your-diabetes-risk-score | 3201 | Govern | USA |
| https://www.beaumont.org/health-wellness/blogs/what-to-know-about-type-1-type-2-diabetes | 3389 | Nonprofit | USA |
| https://www.hackensackmeridianhealth.org/en/HealthU/2021/12/14/Can-You-Get-Diabetes-from-Eating-Too-Much-Sugar | 4124 | Nonprofit | USA |
| https://www.endocrineweb.com/conditions/type-2-diabetes | 4600 | Business | Int |
| https://www.kdhe.ks.gov/904/Take-Control | 5002 | Govern | USA |
| https://www.rwjbh.org/blog/2022/march/is-your-child-at-risk-for-type-2-diabetes-/ | 5088 | Business | USA |
| https://www.dukehealth.org/blog/5-common-myths-about-type-2-diabetes | 5282 | Nonprofit | USA |
| https://www.nidirect.gov.uk/news/preventing-diabetes-and-recognising-its-symptoms | 5845 | Govern | GBR |
| https://ada.com/conditions/diabetes/ | 5850 | Business | DEU |
| https://www.endocrine.org/patient-engagement/endocrine-library/diabetes-and-endocrine-function | 6013 | Nonprofit | USA |
| https://www.texashealth.org/areyouawellbeing/Diabetes/What-is-Prediabetes-and-What-Does-it-Mean-for-Your-Diabetes-Risk | 6175 | Nonprofit | USA |
| https://blog.bonsecours.com/healthy/prediabetes-risk-factors-warning-signs/ | 6680 | Business | USA |
| https://www.sparrow.org/departments-conditions/conditions/type-2-diabetes-children | 7519 | Nonprofit | USA |
| https://healthmatch.io/type-2-diabetes/what-is-the-leading-caUSAe-of-type-2-diabetes | 8584 | Business | USA |
| https://www.tgh.org/institutes-and-services/conditions/type-2-diabetes | 12837 | Business | USA |
| https://www.health.gov.au/health-topics/chronic-conditions/what-were-doing-about-chronic-conditions/what-were-doing-about-diabetes | 13782 | Govern | AUS |
| https://www.renown.org/blog/type-2-diabetes-what-you-should-know | 17716 | Nonprofit | USA |
| https://goforward.com/blog/physical-health/understanding-type-2-diabetes | 18190 | Business | USA |
| https://www.healthnavigator.org.nz/healthy-living/p/preventing-type-2-diabetes/ | 21902 | Govern | NZL |
| https://www.riversidemedicalclinic.com/diabetes-on-the-rise-prevention-strategies/ | 34198 | Business | USA |
| https://www.breathewellbeing.in/blog/caUSAes-of-diabetes/ | 35330 | Business | IND |
| https://elsevier.health/en-USA/preview/screening-for-type-2-diabetes | 52199 | Business | Int |
| https://www.ageuk.org.uk/information-advice/health-wellbeing/conditions-illnesses/diabetes/5-ways-to-cut-your-risk-of-diabetes/ | 52404 | Nonprofit | GBR |
| https://www.centrastate.com/services/diabetes/ | 59070 | Business | USA |
| https://phablecare.com/ailment/diabetes/what-is-type2-diabetes/ | 63819 | Business | IND |
| https://www.idf.org/aboutdiabetes/what-is-diabetes.html | 82334 | Nonprofit | Int |
| https://svhealthcare.org/Wellness-Connection/understanding-diabetes-risk | 92130 | Nonprofit | USA |
| https://www.sugarfit.com/blog/signs-and-symptoms-of-type-2-diabetes | 99795 | Business | USA |
| https://parklandhealthplan.com/living-well/blog/articles/how-to-prevent-type-2-diabetes-in-children-and-teens/ | 120316 | Business | USA |
| https://www.sunlife.ca/en/tools-and-resources/health-and-wellness/preventing-and-treating-illness/what-you-need-to-know-about-prediabetes/ | 144000 | Business | CAN |
| https://www.healthandharmonyrx.com/diabetes.php | 170000 | Business | USA |
| https://www.brownandtoland.com/blog/prediabetes-chance-prevent-type-2-diabetes/ | 175168 | Business | USA |
| https://cecilcountyhealth.org/resources/health-advisory-committee/healthy-lifestyles-task-force/beat-diabetes-with-the-prevent-t2-program/ | 361991 | Govern | USA |
| https://www.holyokepediatrics.com/blog/399558-know-the-warning-signs-of-prediabetes | 381405 | Business | USA |
| https://www.losalamosmedicalcenter.com/news/diabetes-what-you-need-to-know | 394209 | Business | USA |
| https://www.theosborn.org/blog/2021/11/15/pre-conditions-reducing-risk-type-diabetes-09119 | 395717 | Nonprofit | USA |
| https://www.tcrh.org/news/diabetes-what-you-need-to-know | 414205 | Business | USA |
| https://26health.org/heres-why-hispanic-americans-are-at-higher-risk-for-type-2-diabetes/ | 511927 | Nonprofit | USA |
| https://nkfm.org/conditions/diabetes/ | 1345021 | Nonprofit | USA |
| https://inquestmed.com/articles/why-type-2-diabetes-is-dangeroUSA/ | 4082841 | Business | USA |
| https://springtimenutrition.com/type-2-diabetes-risk-factors-what-you-need-to-know/ | 6774214 | Business | USA |
